# Potential Mechanism Prediction of Herbal Medicine for Pulmonary Fibrosis Associated with SARS-CoV-2 Infection Based on Network Analysis and Molecular Docking

**DOI:** 10.3389/fphar.2021.602218

**Published:** 2021-04-12

**Authors:** De Jin, Xuedong An, Yuqing Zhang, Shenghui Zhao, Liyun Duan, Yingying Duan, Fengmei Lian, Xiaolin Tong

**Affiliations:** ^1^ Guang’anmen Hospital, China Academy of Chinese Medical Sciences, Beijing, China; ^2^ Graduate School, Beijing University of Chinese Medicine, Beijing, China

**Keywords:** network pharmacology, molecular mechanisms, pulmonary fibrosis, SARS-CoV-2, Chinese herbal medicine

## Abstract

**Background:** Coronavirus Disease 2019 (COVID-19) is still a relevant global problem. Although some patients have recovered from COVID-19, the sequalae to the SARS-CoV-2 infection may include pulmonary fibrosis, which may contribute to considerable economic burden and health-care challenges. Convalescent Chinese Prescription (CCP) has been widely used during the COVID-19 recovery period for patients who were at high risk of pulmonary fibrosis and is recommended by the Diagnosis and Treatment Protocol for COVID-19 (Trial Version sixth, seventh). However, its underlying mechanism is still unclear.

**Methods:** In this study, an integrated pharmacology approach was implemented, which involved evaluation of absorption, distribution, metabolism and excretion of CCP, data mining of the disease targets, protein-protein interaction (PPI) network construction, and analysis, enrichment analysis, and molecular docking simulation, to predict the bioactive components, potential targets, and molecular mechanism of CCP for pulmonary fibrosis associated with SARS-CoV-2 infection.

**Results:** The active compound of CCP and the candidate targets, including pulmonary fibrosis targets, were obtained through database mining. The Drug-Disease network was constructed. Sixty-five key targets were identified by topological analysis. The findings of Gene Ontology (GO) terms and Kyoto Encyclopedia of Genes and Genomes (KEGG) pathway annotation suggested that the VEGF, Toll-like 4 receptor, MAPK signaling pathway, and TGF-β1 signaling pathways may be involved in pulmonary fibrosis. In the molecular docking analyses, VEGF, TNF-α, IL-6, MMP9 exhibited good binding activity. Findings from our study indicated that CCP could inhibit the expression of *VEGF, TNF-α, IL-6, MMP9, TGF-β1* via the VEGF, Toll-like 4 receptor, MAPK, and TGF-β1 signaling pathways.

**Conclusion:** Potential mechanisms involved in CCP treatment for COVID-19 pulmonary fibrosis associated with SARS-CoV-2 infection involves multiple components and multiple target points as well as multiple pathways. These findings may offer a profile for further investigations of the anti-fibrotic mechanism of CCP.

## Introduction

Severe acute respiratory syndrome coronavirus 2 (SARS-CoV-2) is a newly discovered coronavirus responsible for COVID-19, which causes atypical pneumonia progressing to acute respiratory distress syndrome (ARDS) and acute lung injury (Marini and Gattinoni, 2020). After SARS-CoV-2 infection, patients who have experienced and survived the COVID-19 outbreak may face a greater risk of developing pulmonary fibrosis (PF), which is a chronic, severe, and progressive interstitial lung disease (George et al., 2020). A meta-analysis demonstrated that there was a clear association between the development of PF and respiratory viral infection (Hutchinson et al., 2015). A well-known mechanism is that SARS-CoV-2 invades host cells and interacts with ACE2, which is highly expressed in pneumocytes type II cells and is directly involved in the initiation and progression of inflammation and fibrosis (Ksiazek et al., 2003; Li et al., 2003; Xu et al., 2020). Importantly, PF not only forms as a sequelae to chronic inflammation but is also is genetically influenced by an age-related fibroproliferative process such as idiopathic PF. Further, PF is a well-recognized sequela of ARDS (Burnham et al., 2014). Existing data show that about 40% of COVID-19 patients may develop ARDS, which accounts for a high percentage of COVID-19 patients (Wu et al., 2020). Although in most patients, the virus in patients recovering from COVID-19 has been eradicated, this does not prevent the development of PF. Given these observations, PF after recovery from COVID-19 may result in a substantial medical burden and health-care challenges. Therefore, preventing PF in patients recovering from SARS-CoV-2 infection is an urgent issue that needs to be addressed.

Currently, there are few anti-fibrotic drugs available with clinically positive results, and a limited number of such agents are under investigation (Canestaro et al., 2016). Although the antifibrotic medicines, such as pirfenidone and nintedanib approved by FDA, could delay the decline in lung function, these drugs may not improve the quality of life (QoL) or reduce early mortality (Rochwerg et al., 2016; Brown et al., 2020). More importantly, these drugs may not be prescribed for severe or critical cases with COVID-19 on mechanical ventilation due to oral use only. Moreover, pirfenidone and nintedanib are associated with a high incidence of drug-related side effects such as abnormal liver function and uncomfortable clinical manifestations such as gastrointestinal disturbances and skin reactions (King et al., 2014; Kim and Keating, 2015; Noble et al., 2016; Hanta et al., 2019), which have restricted their clinical application. Thus, the development of an effective therapeutic strategy for PF is urgent.

Traditional Chinese medicines (TCM) have been widely used to treat lung diseases. Recently, several meta-analyses have shown that TCM exerted positive effects on PF, such as delaying the decline of pulmonary function and improving the QoL, with a good safety profile (Liu et al., 2018; Ji et al., 2020). In addition, several pharmacological studies are investigating TCM as an anti-PF treatment. Some studies have revealed that TCM could effectively resist oxidative lesion, histopathological damage (Sun et al., 2018), and reverse extracellular matrix (ECM) as well as Lox2 proliferation by modulating MAPK activation and suppressing the TGF-β/Smad pathway (Tao et al., 2017). A convalescent Chinese prescription (CCP) was widely used for patients with COVID-19 who were in the recovery period and were at high-risk of pulmonary fibrosis, as recommended by the Diagnosis and Treatment Protocol for COVID-19 (Trial Version sixth, seventh) published by the National Health Commission of the People’s Republic of China (National Health Commission of the People’s Republic of China, 2020a; National Health Commission of the People’s Republic of China, 2020b).

**FIGURE 1:**
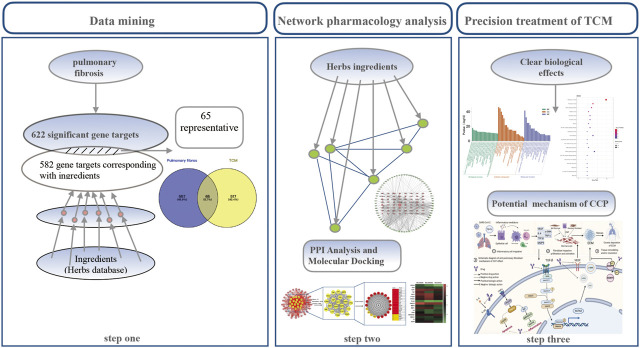
Schematic diagram of the integrated pharmacology strategy approach that combines quantitative analysis of components, network analysis, and molecular docking to investigate the mechanisms of Convalescent Chinese prescription (CCP) treatment against pulmonary fibrosis.

Although clear clinical benefits exist, very little has been elucidated about the potential molecular mechanism involved. Network pharmacology is a branch of pharmacology that uses network methods to analyze the synergistic relationship among drugs and diseases and targets via “multi-component, multi-target, multi-pathway” analyses, and can build a multi-dimensional network model of “drug–component–target–disease” to explore the relationship between drugs and diseases (Missiuro et al., 2009; von Mering et al., 2003). This study was based on network pharmacology and systematically analyzed the effective ingredients, potential targets, pathways, and biological processes of CCP used during the recovery period of COVID-19. The study screened the main active ingredients of CCP via a molecular docking approach to explore the potential molecular mechanisms of action involved in CCP interference with PF associated with SARS-CoV-2 infection. The flowchart of the whole study design is illustrated in Figure 1.

## Materials and Methods

### Active Components Database

CCP contains 18 types of Chinese herbal medicine (Table 1). All ingredients related to CCP were screened by the Traditional Chinese Medicine Systems Pharmacology database and Analysis Platform (TCMSP), PubChem database (http://pubchem.ncbi.nlm.nih.gov), China Knowledge Network, PubMed and BATMAN-TCM) and Shanghai Institute of Organic Chemistry of CAS. Chemistry Database [DB/OL].

**TABLE 1:** Composition of herbs in Convalescent Chinese prescription (CCP).

Latin name	English name	Chinese name	Abbreviation
Radix astragali seu hedysari	Milkvetch root	Huang qi	HQ
Radix codonopsis	Tangshen	Dang sheng	DS
Rhizoma atractylodis	Largehead atractylodes rhizome	Bai zhu	BZ
Macrocephalae			
Radix adenophorae	Fourleaf ladybell root	Nan sha shen	NSS
Radix glehniae	Coastal glehnia root	Bei sha shen	BSS
Pericarpium citri reticulatae	Dried tangerine peel	Chen pi	CP
Poria	Lndian bread	Fu ling	FL
Radix ophiopogonis	Dwarf lilyturf tuber	Mai dong	MD
Radix salviae miltiorrhizae	Radix salviae miltiorrhizae	Dan shen	DSH
Bulbus fritillariae thunbergii	Thunberbg fritillary bulb	Zhe bei mu	ZBM
Hirudo	Leech	Shui zhi	SZ
Fructus crataegi	Hawthorn fruit	Chao Shan zha	CSZ
Massa medicata fermentata	Medicated leaven	Shen qu	SQ
Fructus hordei germinatus	Germinated barley	Mai ya	MY
Rhizoma dioscoreae	Common yam rhizome	Shan yao	SY
Eupolyphaga seu steleophaga	Ground beetle	Tu bie chong	TBC
Liquorice root	Radix glycyrrhizae	Gan cao	GC
Rhizoma pinelliae	Pinellia tuber	Ban xia	BX

### Screening of Active Ingredients

The critical parameters of oral bioavailability (OB), drug-likeness (DL), and drug half-life (HL) were used to screen the active components of CCP. OB is an essential indicator for objective evaluation of the internal quality of drugs. OB defines the percentage of an orally administered dose of unchanged drug that reaches the systemic circulation and represents the convergence of the absorption, distribution, metabolism, and excretion (ADME) process. High OB is often a key indicator to determine the “drug-like” properties of bioactive molecules as therapeutic agents. Molecules with OB >30% were considered to have good OB in the present study (Xu et al., 2012). DL is a qualitative concept used in drug design to estimate the drug-like properties of a prospective compound and helps to optimize pharmacokinetics and pharmaceutical properties, such as solubility and chemical stability. The “drug-like” level of the compounds was set at 0.18, which is used as a selection criterion for the “drug-like” compounds for traditional Chinese herbs (Tao et al., 2013); thus, ingredients with DL > 0.18 were selected. The HL (t1/2) means that the time it takes to reduce the number of compounds in the body by half, is arguably the most important property of an active ingredient as it dictates the timescale over which the compound may elicit therapeutic activity (Ma et al., 2018). HL values > 4 h were selected.

### Drug and Disease Target Fishing

We screened for potential targets of the herbs constituting CCP in the TCMSP database. If there was no corresponding drug target in the database, we determined the molecular structural formula based on available chemical formulas from the literature and predicted their potential targets based on the spatial structure of the molecular structure formula in the Swiss-target prediction. The final potential targets of these herbs were obtained by screening the corresponding drug targets for constituents and removing duplicates. The qualitative targets were matched to the UniProt database for normalization (Missiuro et al., 2009). In this study, PF was considered a phenotype in a convalescent patient with SARS-CoV-2 infection. Therefore, the targets related to pulmonary fibrosis were explored based on the OMIM database, drug bank database, and the DisGeNET database. We merged all queried targets and eliminated duplicated results. Finally, standardized names were implemented via the UniProt database.

### Network Construction and Analysis

To further explore the mechanisms of CCP's treatment effects on PF of patients with COVID-19 during the rehabilitation stage, we established a network drug-disease map to show the association between the active components in the CCP and its potential targets using Cytoscape v3.7.1. The components and targets were represented by triangles and circles, respectively, and the interaction between the two was shown by a connecting line. Overlapping portions between drug targets and disease targets are represented by Wayne's diagram. To explain the interaction between target proteins, overlapping target proteins between CCP and PF were uploaded to STRING to obtain information on protein-protein interaction (PPI) (Mering et al., 2003). We selected a medium confidence data threshold of >0.4. The obtained protein-protein interaction data were submitted to Cytoscape 3.7.1 to build a PPI network. Additionally, the Significant Gene Ontology (GO) Pathway and the Kyoto Encyclopedia of Genes and Genomes (KEGG) Pathway were screened by the DAVID database.

### Molecular Docking

The above-mentioned PPI analysis generated potential hub genes active in PF treatment. Binding activity between the active drug components and the hub genes were evaluated by flexible molecular docking using Surflex-Dock software (Jain, 2007). Surflex-Dock uses an idealized active site ligand called a prototype molecule as a target for generating a hypothetical conformation of a molecule or a molecular fragment (Jain, 2007). These hypothetical conformations are all scored by the Hammerhead scoring function, which also serves as the objective function for the local optimization of the conformation (Welch et al., 1996). Through the crossover process, a large number of conformations are assembled from the complete molecule to achieve flexible docking. The crystal structure of the target proteins was obtained from the Protein Data Bank (Berman et al., 2003). The structural formula (MOL2 format) of the compounds were available at the TCMSP database and PubChem. If the structural formula was not available, we would manually draw the molecular structure with ChemDraw software. The protein targets were processed by removing water, adding hydrogen, and extracting the ligand structure accordingly and finally, Surflex-Dock v.2.1 was run to perform molecular docking (Lill and Danielson, 2011; Spitzer and Jain, 2012; Yuan et al., 2016). In order to examine the stability of molecular Docking, we also perform another molecular docking (dockthor, https://dockthor.lncc.br/v2/). A similar result occurs if molecular docking is relatively precise.

## Results

### Active Component Screening

The active components of CCP were retrieved from the TCMSP database based on three parameters (OB>30%; DL > 0.18; HL > 4). Ultimately, 308 related components were identified as active ingredients in the CCP. Milkvetch root (HQ, 16 ingredients), Tangshen (DS, 19 ingredients), Largehead atractylodes rhizome (BZ, 5 ingredients), Fourleaf ladybell root (NSS, 5 ingredients), Coastal glehnia root (BSS, 3 ingredients), Dried tangerine peel (CP, 5 ingredients), Indian bread (FL, 14 ingredients), Dwarf lilyturf tuber (MD, 4 ingredients), Radix Salviae Miltiorrhizae (DSH, 52 ingredients), Thunberbg fritillary bulb (ZBM, 4 ingredients), Leech (SZ, 32 ingredients), Hawthorn fruit (CSZ, 7 ingredients), Medicated leaven (SQ, 12 ingredients), Germinated barley (MY, 10 ingredients), Common yam rhizome (SY, 16 ingredients), Ground beetle (TBC, 17 ingredients), Radix glycyrrhiza (GC, 76 ingredients), and Pinellia tuber (BX, 11 ingredients). Leech and Ground beetle were obtained by literature mining via China National Knowledge Infrastructure (CNKI) (Guoqiang et al., 2018; Wu et al., 2018). The potential compounds of the CCP formulation and the respective ADME parameters are shown in detail in Supplementary Material S1.

### Target Fishing for Drug Components and Disease and Establishing the Drug–Disease Target Network

Targets from 14 Chinese herbs were available in the TCMSP database. Targets from Leech (SZ) were available from a previous report (Guoqiang et al., 2018). Targets from Ground beetle (TBC) were available in Swiss-target Prediction software according to its components (Wu et al., 2018). Targets from Dwarf lilyturf tuber (MD) and Hawthorn fruit (CSZ) were available in the Chemistry Database. We matched the components of herbals with the corresponding targets. We obtained 307 targets of HQ, 102 targets of DS, 19 targets of BZ, 39 targets of NSS, 181 targets of BSS, 30 targets of MD, 6 targets of CP, 59 targets of FL, 127 targets of BX, 129 targets of DSH, 33 targets of ZBM, 319 targets of SZ, 307 targets of GC, 73 targets of MY, 209 targets of CSZ, 32 targets of SQ, 96 targets of SY. In order to obtain the targets of TBC, we manually searched the literature to find the components (Wu et al., 2018), submitting components into SwissTargetPredict Tools. We obtained 251 targets of TBC. After eliminating duplicates, the final number of identified targets in CCP was 582. The potential targets in detail are shown in Supplementary Material S2. In total, 622 targets associated with PF were collected through the OMIM, Drugbank, and DisGeNET databases. Details on the targets associated with PF are shown in Supplementary Materials 3**.**

**FIGURE 2:**
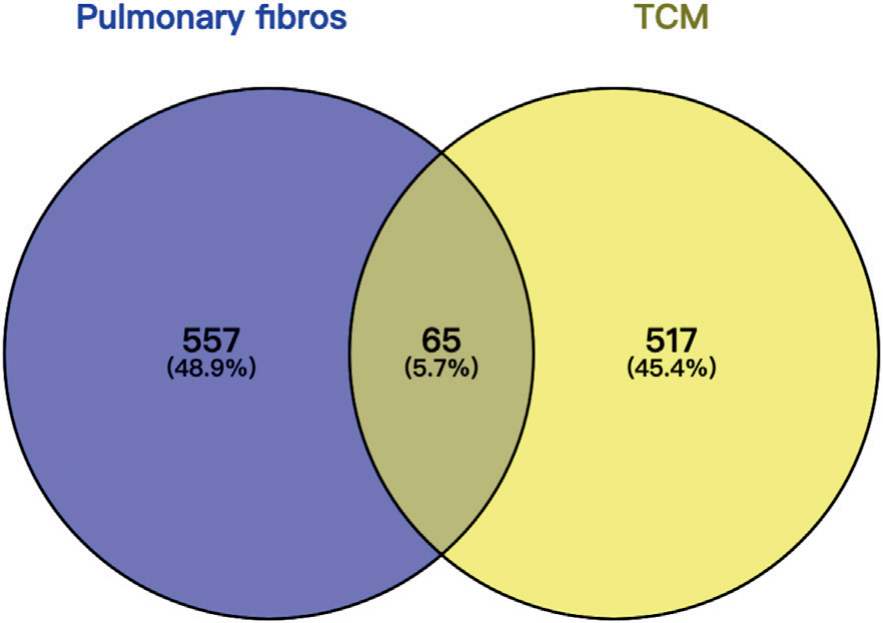
Wayne diagram of commo gene targets of Convalescent Chinese prescription (CCP) drug therapy and pulmonary fibrosis.

**FIGURE 3:**
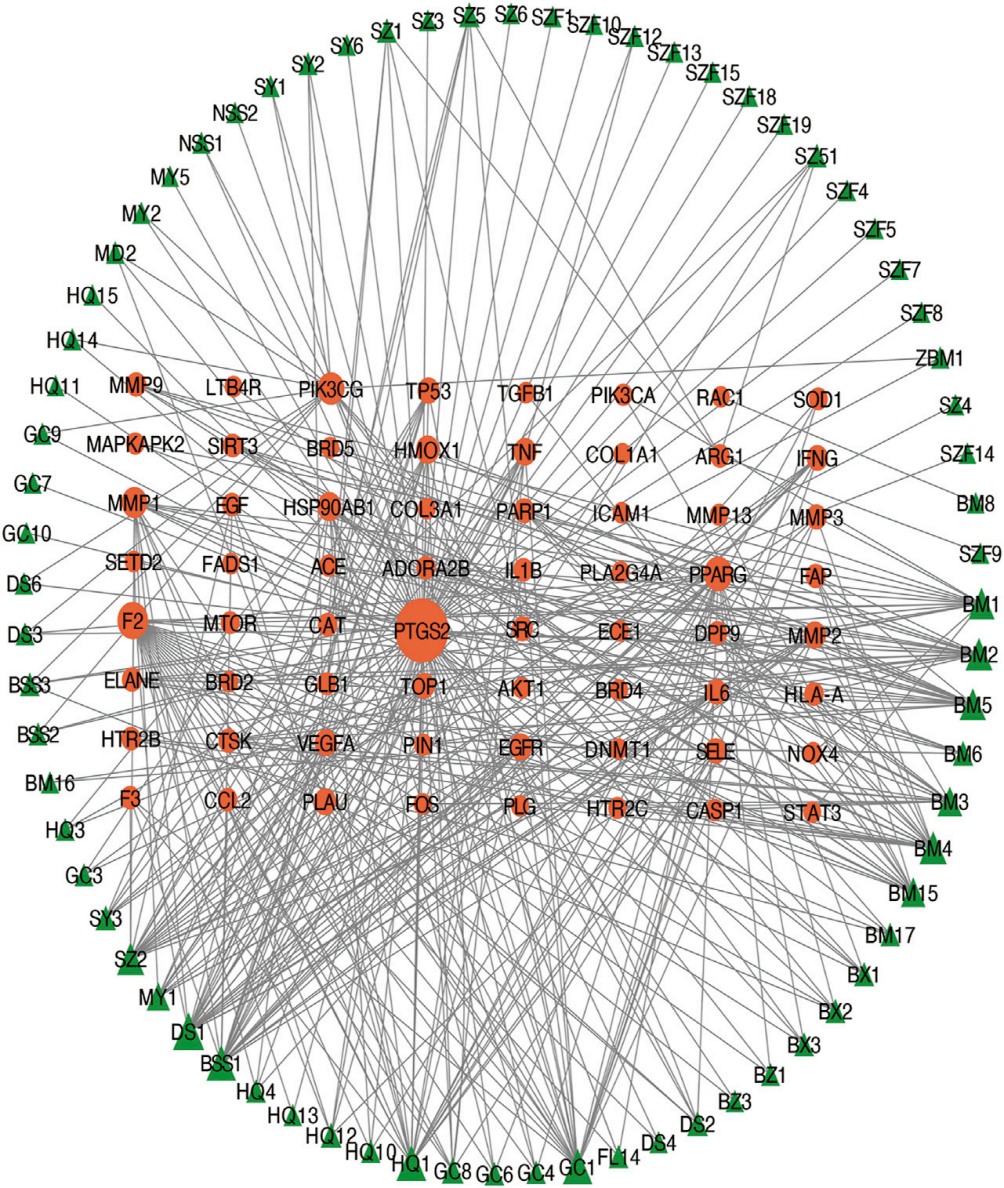
Construction of the drug (herbal ingredients)–disease (pulmonary fibrosis targets) network. The nodes representing drug candidate compounds are shown as green triangles and the targets are indicated by orange circles.

**FIGURE 4:**
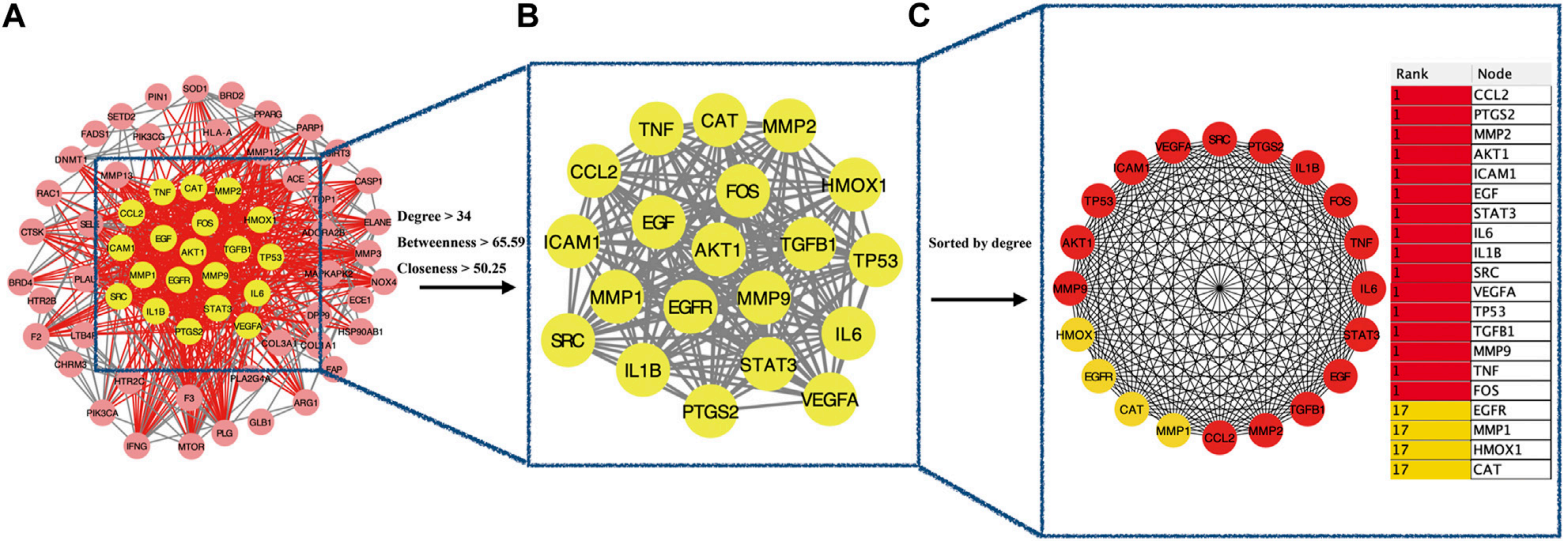
Identification of candidate targets for Convalescent Chinese prescription (CCP) against pulmonary fibrosis via Protein–protein interaction Analysis. **(A)**. Protein–protein interaction (PPI) networks of shared targets between Convalescent Chinese prescription (CCP) and pulmonary fibrosis were analyzed by STRING 11.0. **(B)**. The most significant module identified by the topology selection (degree centrality >34, betweenness centrality >65.59, closeness centrality >50.2). **(C)**. The core 20 targets (hub targets) in the PPI network ranked by degree centrality using the cytoHubba plug-in.

The intersection (65 common targets) between drug targets and disease targets is shown in Figure 2, and the details of the shared targets are shown in Table 2. Subsequently, we mapped the components of CCP based on these 65 targets to construct the drug (components)–disease (PF targets) network shown in Figure 3. Next, we submitted the 65 targets to the string tool to generate the PPI network. The PPI network was also visualized using Cytoscape 3.7.1 software. As shown in Figure 4A, 65 nodes and 683 edges were identified in the PPI network (Network Properties: Degree = 20, Betweenness = 21.65795, Closeness = 40.83333). Subsequently, a topological analysis of the PPI network was implemented using network properties values greater than the median values (Degree = 34, Betweenness = 65.59, Closeness = 50.25), as shown in Figure 4B. The identified targets (*AKT1, TNF, IL6, TP53, VEGFA, IL1B, MMP9, EGFR, CCL2, PTGS2, STAT3, EGF, SRC, MMP2, FOS, CAT, HMOX1, ICAM1, MMP1, TGFB1*) represented potential key targets for the therapeutic effects of CCP (Figure 4C).

**TABLE 2:** Shared hub targets between Convalescent Chinese prescription (CCP) and pulmonary fibrosis.

Number	Gene name	Number	Gene name
1	ADORA2B	37	HTR2C
2	AKT1	38	ICAM1
3	CTSK	39	PLG
4	ELANE	40	SELE
5	F2	41	DNMT1
6	HTR2B	42	SETD2
7	MAPKAPK2	43	SRC
8	MMP1	44	RAC1
9	MMP13	45	TNF
10	MMP2	46	PLAU
11	MMP3	47	IL6
12	MMP9	48	IFNG
13	MTOR	49	CCL2
14	NOX4	50	IL1B
15	PIK3CA	51	F3
16	STAT3	52	SOD1
17	FADS1	53	TP53
18	FAP	54	COL1A1
19	HSP90AB1	55	EGF
20	LTB4R	56	TOP1
21	PARP1	57	COL3A1
22	TGFB1	58	PPARG
23	EGFR	59	PIK3CG
24	VEGFA	60	CHRM3
25	ACE	61	PLA2G4A
26	BRD2	62	CAT
27	BRD4	63	MMP12
28	DPP9	64	ARG1
29	HLA-A	65	GLB1
30	HMOX1		
31	PIN1		
32	PTGS2		
33	SIRT3		
34	CASP1		
35	ECE1		
36	FOS		

### Enrichment Analysis

**FIGURE 5:**
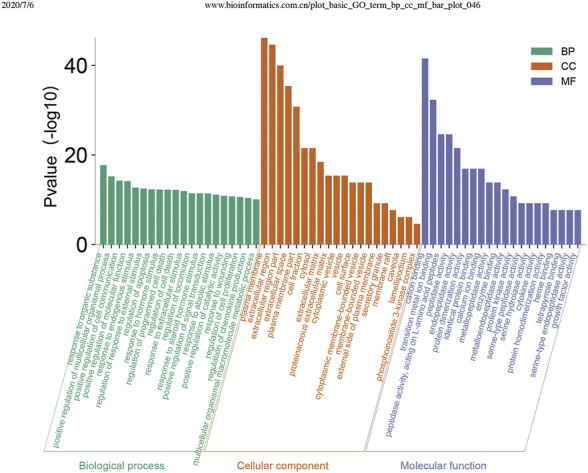
Enrichment analysis of the potential targets of Convalescent Chinese prescription (CCP) against pulmonary fibrosis by R software 3.4.2 for the Gene Ontology database. Top 20 biological process (BP) terms, cellular component (CC) terms, and molecular function (MF) terms are shown as green bars, orange bars, and purple bars, respectively, according to “*p*-value (<0.05), Bonferroni correction.”

Using the DAVID database, GO enrichment analysis yielded GO entries (*p* < 0.05) comprising 336 biological processes (BP), 30 cellular components (CC), and 27 molecular functions (MF). The top 20 entries were selected from BP, CC, and MF, respectively, in order of -lgP value (Figure 5). In the BP, the primary target in the extracellular region was the cytosol; for MF, the targets mainly involved enzyme binding, protein binding, and identity protein binding, and cytokine activity.

**FIGURE 6:**
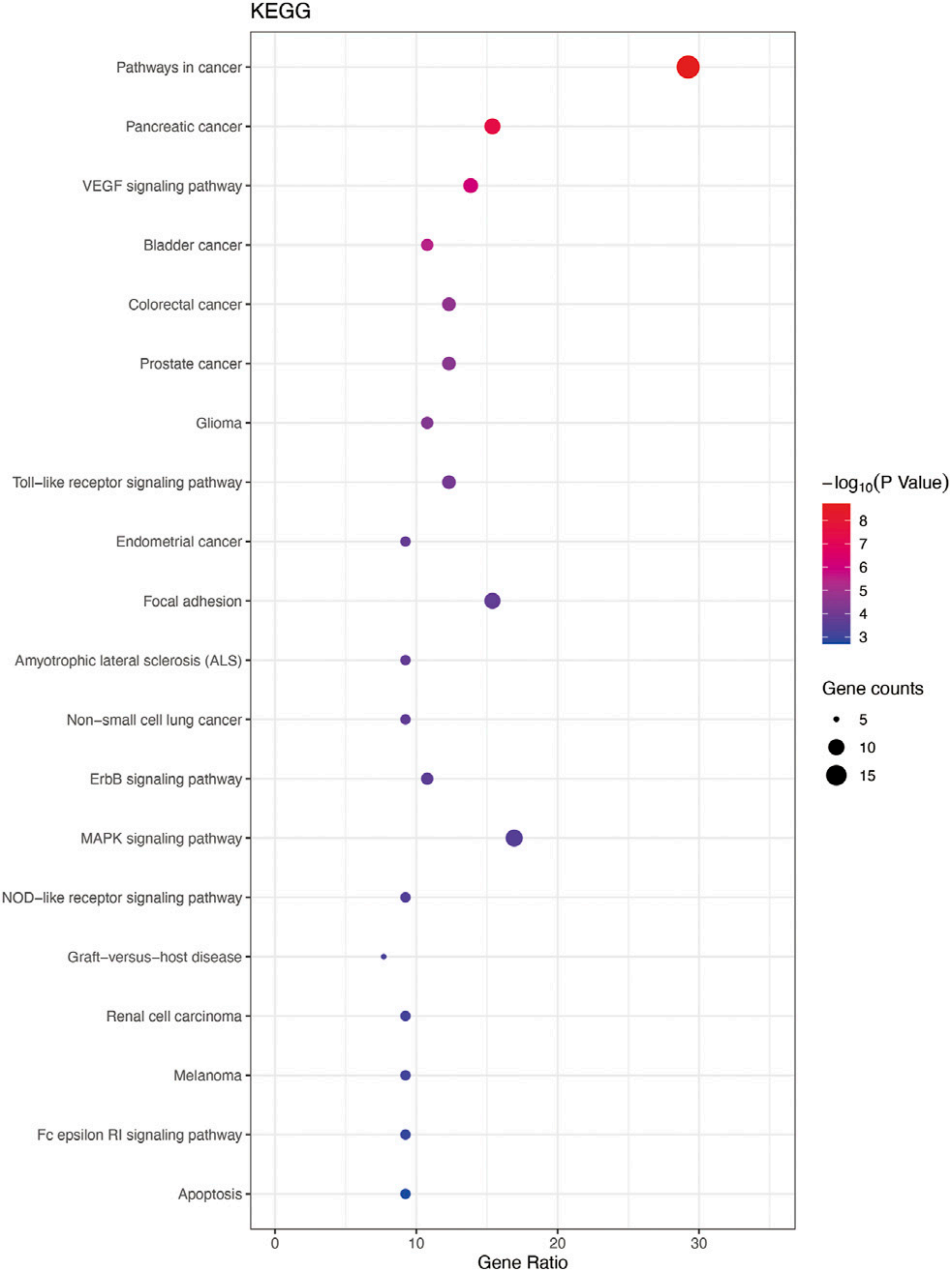
Enrichment analysis for KEGG. Top 20 KEGG pathways listed by bubble chart according to the “*p*-value (<0.05), Bonferroni correction.”

In total, 93 terms (*p* < 0.05) were obtained from the KEGG pathway enrichment analysis using DAVID data. The first 20 entries were selected according to the–lgP value to draw a bubble diagram (Figure 6). The main pathways included the VEGF, Toll-like 4 receptor, mitogen-activated protein kinase family (MAPK), NOD−like receptor signaling pathways. Targets involved in the signaling pathways were as follows: VEGF pathway (*PIK3CG, AKT1, PLA2G4A, PTGS2, VEGFA, RAC1, PIK3CA, MAPKAPK2, SRC*); Toll-like 4 receptor pathway (*PIK3CG, AKT1, FOS, IL6, TNF, RAC1, IL1B, PIK3CA*); MAPK pathway (*EGFR, AKT1, FOS, PLA2G4A, TNF, RAC1, TP53, IL1B, MAPKAPK2, EGF, TGFB1*); and in the NOD-like receptor pathway (*HSP90AB1, IL6, TNF, CCL2, IL1B, CASP1*).

### Molecular Docking

**FIGURE 7:**
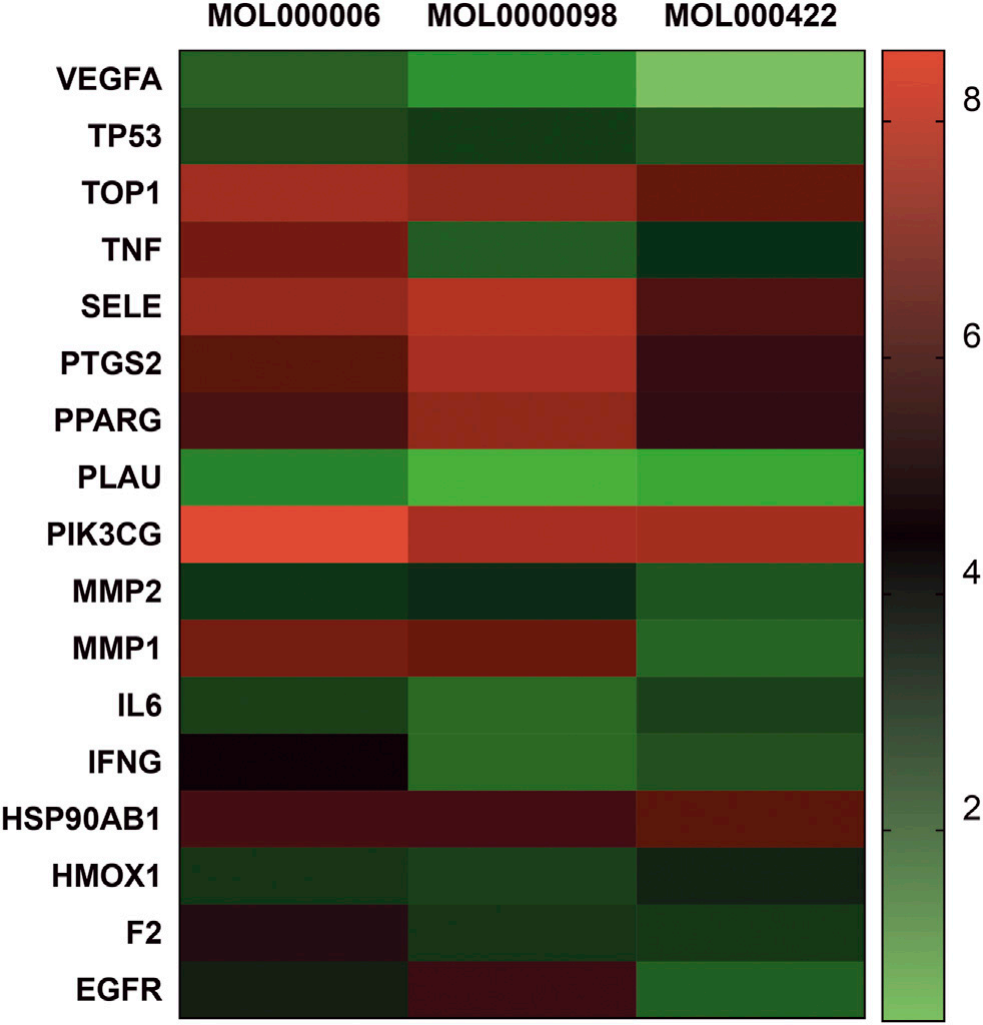
Heat map of Molecular docking. Molecular models of the binding of quercetin (MOL0000098), kaempferol (MOL000422), luteolin (MOL000006) with TOP, MMP2, MMP9, IFNG, SELE, PLAU, VEGFA, HMOX1, F2, TNF, TP53, PPARG, PIK3CG, IL6, PTGS2, HSP90AB1, and EGFR, respectively.

The binding ability and of herbal components to core protein targets were validated by molecular simulations. Using molecular docking by Surflex-Dock modeling, a docking score greater than 3 was considered as a stable compound binding to the protein. In this respect, the top 20 nodes were selected from the drug-disease network (Figure 3) according to the degree value. They were as follows: TOP1 (PDB ID: 1K4t), MMP2 (PDB ID: 1cxw), MMP9 (PDB ID:1eak), IFNG (PDB ID: 1fyh), SELE (PDB ID: 1git), PLAU (PDB ID:1kdu), VEGFA (PDB ID: 1kmx), HMOX1 (PDB ID: 1ni6), F2 (PDB ID: 1nL1), TNF (PDB ID: 2e7a), TP53 (PDB ID: 2k8f), PPARG (PDB ID: 3e00), PIK3CG (PDB ID: 3I13), IL6 (PDB ID: 4cni), PTGS2 (PDB ID: 5f19), HSP90AB1 (PDB ID: 5ucj), EGFR (PDB ID: 5wb7), quercetin (MOL0000098), kaempferol (MOL000422), and luteolin (MOL000006). As shown in Figure 7, quercetin, kaempferol, and luteolin exhibited high binding activity to targets associated with PF. for example IL-6 (score = 3.0236, 3.6316, 3.7055, respectively), TNF-α (score = 3.2116, 3.9889, 5.9409, respectively), VEGF (score = 3.0175, 3.844, 3.1564, respectively), MMP9 (score = 5.7384, 3.079, 5.9618, respectively). Detailed blinding scores were shown in the Heat map in Figure 7 and in the Supplementary Material S4. Moreover, another molecular docking presents a similar outcome by dockthor. The molecular docking gives higher reliability of obtained results in this work. Detailed blinding scores in dockthor were shown in Supplementary Material S5.

## Discussion

CCP has been described as a suitable treatment for the recovery phase of patients with COVID-19. In this study, a total of 116 active ingredients and 583 action targets of CCP were screened based on the network pharmacology approach. By further screening, 72 core compounds and 26 key action targets were obtained. Network pharmacology analysis embodies the holistic and correlative characteristics of the combined action of multiple components and targets of traditional Chinese medicine. According to the topological property analysis of the "drug-disease" network, the top 5 key compounds in CCP are quercetin, kaempferol, luteolin, TBC2, and TBC4. In addition, other the components were collected through multiple databases such as China Knowledge, PubMed, and BATMAN-TCM. The chemical constituents (TBC2, TBC4, TBC5) are not included in the TCMSP platform. The targets of these components need to be further studied. Quercetin is known to possess marked antioxidative, anti-inflammatory, and antifibrotic capacities. Dietary quercetin supplementation also decreases chronic systemic inflammation (Stewart et al., 2008). Quercetin can reduce the expression of transforming growth factor-β1 (*TGF-β1*), *α*-smooth muscle actin (*α-SMA*), and tumor necrosis factor-α (*TNF-α*), inhibit alveolar cell apoptosis and reduce lung tissue inflammation and fibrosis injury in rats, which can effectively improve lung fibrosis (Ma et al., 2018; Zhang et al., 2018; Wei et al., 2019). Kaempferol, a polyphenol with potent antioxidant activity, is an ester of caffeic acid and quinic acid (Nardini et al., 2002). Luteolin exhibits various pharmacological activities, including antioxidative, antiviral, antibacterial, anti-inflammatory, anti-endotoxin, antitumor, and liver-protective effects (Rohit and Mohan Rao, 2013; Tajik et al., 2017; Wei et al., 2017).

According to the results of the PPI network analysis and the topological property analysis of the "Drug–disease" network, the targets of action of CCP were the cytokine IL-6, members of the MAPK family, and PTGSE or prostaglandin G/H synthase. The results of GO functional enrichment analysis revealed that CCP components were mainly involved in the cell communication, endogenous stimulus regulation, apoptosis, programmed cell death, steroid hormone stimulus, signal transduction, regulation of catalytic activity, wounding regulation of cell proliferation, regulation of chemokine production and multicellular organismal macromolecule metabolic process. The KEGG pathway enrichment mainly involved Toll-like 4 receptor signaling and immune response pathways. Additionally, the NOD-like receptor signaling pathway and the Graft-versus-host disease pathway involved the *IL-6* gene. IL-6 is a complex ∼25-kDa cytokine, acting in both pro- and anti-inflammatory capacities (Weidhase et al., 2019). Importantly, IL-6 trans-signaling via IL-6/soluble IL-6 receptor (sIL-6R) complexes, but not classic signaling via IL-6/membrane bound IL-6 receptor (IL-6R) complexes, has been shown to prevent the apoptosis of T cells and promote tissue damage (Sommer et al., 2014).

The results of this study suggested that CCP may have an interventional effect on viral infections and lung injury. The pleotropic cytokine, TNF-α, plays a significant role in the pathogens of chronic inflammatory diseases. The MAPK signaling pathway controls diverse cellular processes in response to a variety of extracellular stimuli (Kiss et al., 2019). Taken together, the results suggest that CCP may play a role in the recovery period of COVID-19 by exerting antiviral, bacteriostatic, anti-inflammatory, and immunomodulatory activity. Chinese medicine has always been known for its theoretical understanding and clinical practice of “preventing diseases before disease onset.” COVID-19 has a relatively long disease duration, and the recovery period is characterized by a state of low immunity. This study revealed that CCP could regulate immune function through multiple pathways and multiple targets.

We then performed docking studies for TOP, MMP2, MMP9, IFNG, SELE, PLAU, VEGFA, HMOX1, F2, TNF, TP53, PPARG, PIK3CG, IL6, PTGS2, HSP90AB1, EGFR, using the critical ingredients quercetin, kaempferol, and luteolin as ligands. Molecular docking is used to evaluate whether ligands and proteins may bind thermodynamically. Overall, the scores of the active compounds with the key targets were for the majority positive with scores greater than 3, demonstrating that quercetin, kaempferol, and luteolin exhibited good binding properties with VEGFA, TNF, MMP9, and IL-6, respectively, all of which play an essential role in PF.

The pathological process of PF could be roughly divided into three stages. The first stage involves the diffuse damage of vascular endothelial cells and alveolar epithelial cells by pathogenic factors, which initiates the inflammatory immune response. Second, inflammatory cells release a variety of cytokines and inflammatory mediators, expanding tissue damage and causing interstitial hyperplasia. The third step involves the migration and proliferation of fibroblasts and endothelial cells and the metabolic disorders of collagen and other ECM components, which further aggravate inflammatory damage and proliferation in a positive feedback manner. Eventually, the process could lead to the replacement and reconstruction of normal lung tissue. These three processes exist simultaneously, which are interrelated and interact (Fernandez and Eickelberg, 2012; Todd et al., 2012; Kolahian et al., 2016).

**FIGURE 8:**
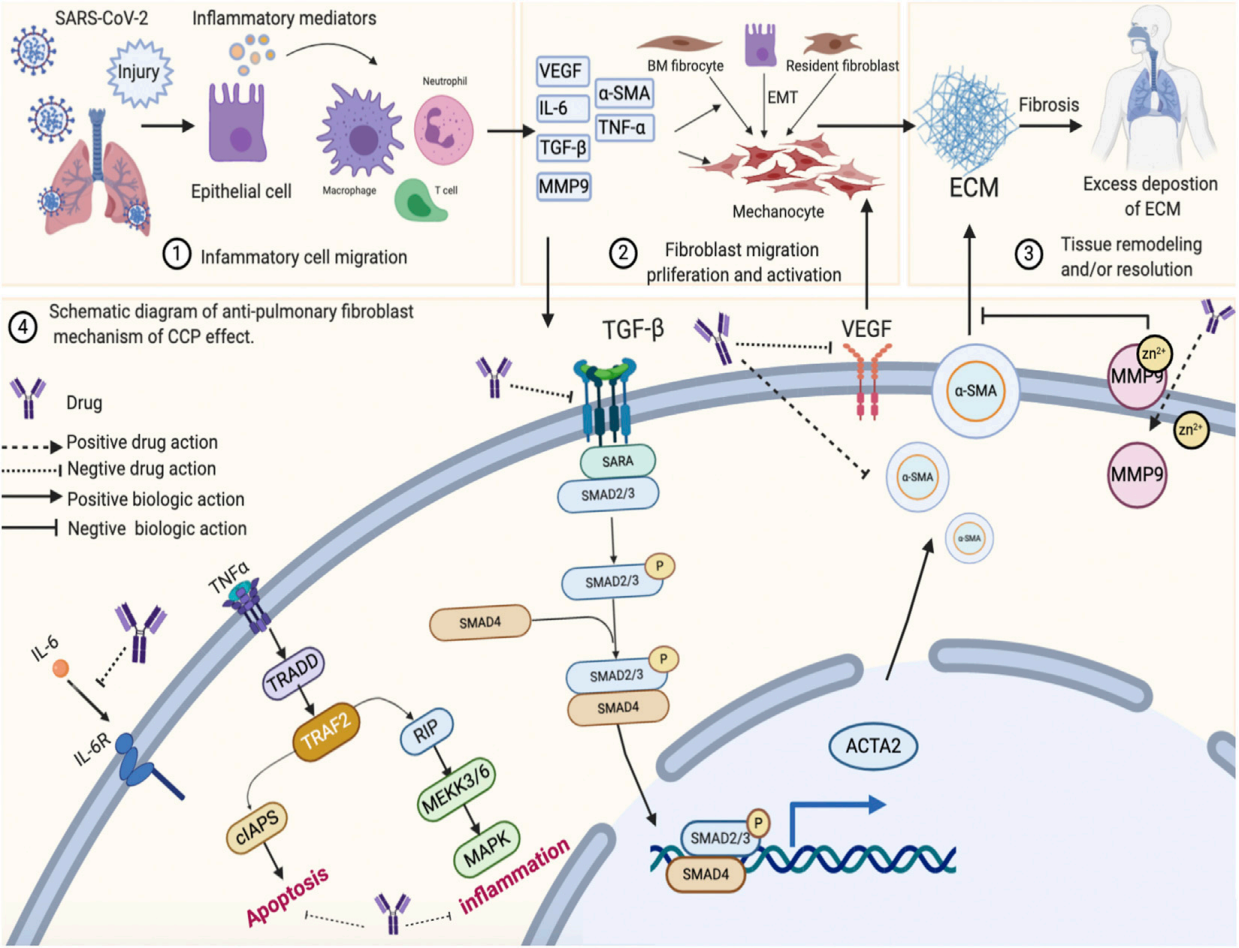
The schematic diagram illustrating the proposed activity model of Convalescent Chinese prescription (CCP) in pulmonary fibrosis. Exposure to SARS-CoV-2 infection damages lung epithelial cell, resulting in injury or cell death and the release of inflammatory mediators into the extracellular space. This signal is recognized by Macrophages cell, T cell, Neutrophils cell. Activation of these cells leads to inflammatory responses in lung epithelial cell. Lung injury also induces fibroblast migration proliferation and activation. These processes result in tissue remodeling or resolution and excess deposition of extracellular matrix (ECM). Chronic, unresolved lung inflammation owing to the activation of various signaling pathways (such as TNFa, MAPK and TGFβ) eventually results in progressive lung fibrosis.

VEGF-A is considered a critical factor in the pathogenesis of PF (Medford and Millar, 2006; Barratt et al., 2014). VEGFR is a functional receptor of VEGF and an important target for mediating angiogenesis, which stimulates the proliferation and aggregation of fibroblasts, promotes epithelial-mesenchymal transition, activates multiple abnormal signaling pathways, further induces the formation of fibroblast foci, and causes the activation of the MMP family members to destroy the alveolar structure, promote matrix deposition, and induce scar formation (Leung et al., 1989; Ferrara et al., 2003; Bates, 2010). This process generates other mediators involved in the inflammatory response, such as TNF-α and IL-6, which directly or indirectly promote the synthesis of the ECM through interaction with other cytokines (Le et al., 2014; Hou et al., 2018; Papiris et al., 2018). More importantly, several studies have robustly documented that silencing the expression of TGF-β1 reduces inflammation and slows the progression of PF (Baowen et al., 2010; Liu et al., 2013; Peng et al., 2013), which play a pivotal role in PF (Hu et al., 2018; Li et al., 2018). Findings from our study indicated that CCP could inhibit the expression of *VEGF, TNF-α, IL-6, MMP9*, and *TGF-β1* via the VEGF, Toll-like 4 receptor, MAPK, and TGF-β1 signaling pathways. The potential mechanisms involved in CCP activity are summarized in Figure 8.

## Conclusion

The findings suggest that CCP treatment of COVID-19 PF associated with SARS-CoV-2 infection involves multiple components and multiple target points as well as multiple pathways. Such proteins should be interesting to future studies that provide a novel direction for the mechanisms of PF associated with SARS-CoV-2 infection development and a new intervention target for clinical investigations, covering these gaps in research to be able to draw more meaningful conclusions about the benefits of CCP. These findings may offer a rationale for further investigations of the anti-fibrotic mechanisms of CCP therapy.

## Data Availability

The original contributions presented in the study are included in the article/Supplementary Material, further inquiries can be directed to the corresponding authors.
